# *Pinus thunbergii* Parl. Somatic Plants’ Resistance to *Bursaphelenchus xylophilus* Depends on Pathogen-Induced Differential Transcriptomic Responses

**DOI:** 10.3390/ijms25105156

**Published:** 2024-05-09

**Authors:** Tingyu Sun, Yahui Wang, Xiaoqin Wu, Yang Wang, Aixia Yang, Jianren Ye

**Affiliations:** 1 College of Forestry, Nanjing Forestry University, Nanjing 210037, China; suntingyunjfu@163.com (T.S.);; 2 State Key Laboratory of Horticultural Crop Germplasm Resources Creation, Utilization of Ministry of Agriculture and Rural Affairs, Institute of Horticulture Research, Anhui Academy of Agricultural Sciences, Hefei 230041, China; 3 Collaborative Innovation Center of Sustainable Forestry in Southern China, Nanjing 210037, China; 4 Institude of Forest Pest Control, Jiangxi Academy of Forestry, Nanchang 330032, China

**Keywords:** nematode-resistant clone, *Pinus thunbergii*, pine wilt disease, somatic plants, transcriptome

## Abstract

*Pinus thunbergii* Parl. is an economically and medicinally important plant, as well as a world-renowned horticultural species of the *Pinus* genus. Pine wilt disease is a dangerous condition that affects *P. thunbergii*. However, understanding of the genetics underlying resistance to this disease is poor. Our findings reveal that *P. thunbergii*’s resistance mechanism is based on differential transcriptome responses generated by the early presence of the pathogen *Bursaphelenchus xylophilus*, also known as the pine wood nematode. A transcriptome analysis (RNA-seq) was performed to examine gene expression in shoot tissues from resistant and susceptible *P. thunbergii* trees. RNA samples were collected from the shoots of inoculated pines throughout the infection phases by the virulent *Bursaphelenchus xylophilus* AMA3 strain. The photosynthesis and plant–pathogen interaction pathways were significantly enriched in the first and third days after infection. Flavonoid biosynthesis was induced in response to late infestation (7 and 14 days post-infestation). *Calmodulin*, *RBOH*, *HLC* protein, *RPS*, *PR1*, and genes implicated in phytohormone crosstalk (e.g., *SGT1*, *MYC2*, *PP2C*, and *ERF1*) showed significant alterations between resistant and susceptible trees. Furthermore, salicylic acid was found to aid pine wood nematodes tolerate adverse conditions and boost reproduction, which may be significant for pine wood nematode colonization within pines. These findings provide new insights into how host defenses overcame pine wood nematode infection in the early stage, which could potentially contribute to the development of novel strategies for the control of pine wilt disease.

## 1. Introduction

*Pinus thunbergii* Parl. is a well-known and popular horticultural tree from the genus *Pinus*. At the same time, it is a valuable economic and therapeutic plant. Wood of the *P. thunbergii* tree is widely utilized in building, furniture, and paper production, and *P. thunbergii* seeds can also be turned into oil [1,2]. Moreover, *P. thunbergii* has traditionally been used for edible and medicinal purposes to treat several disorders, such as neuralgia, acute lung injury, and diabetes [3,4]. As an important commercial pine species, *P. thunbergii*, which is used for coastal reforestation and landscaping, has been extensively cultivated in China for more than 100 years [5]. However, because the *P. thunbergii* species is sensitive to pine wood nematode (PWN), its population has declined substantially since the first report of pine wilt disease (PWD) in Nanjing City, China [6,7].

PWD is a severe forest disease affecting the *Pinus* species, caused by PWN (*Bursaphelenchus xylophilus*) and resulting in the death of a significant number of pine trees [8,9]. PWN is native to North America, where it very rarely kills native pine trees [10]. East Asia was the primarily affected areas, particularly China, Japan, and Korea, where the pathogen continues to cause irreparable damage to forest ecosystems and severe economic losses [11,12,13]. In Europe, the PWN was first detected in 1999, specifically in southwestern Portugal, and had isolated incursions into Spain. Today, it has spread to more than 30% of Portugal, causing extensive damage to Portuguese forests [14,15]. Moreover, the number of damaged conifer trees is increasing every year. Researchers have implemented a series of strategies to combat PWD, including the removal of dead trees, early molecular detection for conifers, and injection of nematocidal substances [16,17,18]. However, these measures only delayed the spread of PWD and cannot completely control the disease [19]. The breeding program for elite nematode-resistant *Pinus* species was an efficient and environmentally friendly approach to controlling PWD [20,21,22]. Somatic embryogenesis was the most promising technique for mass propagation of selected elite conifers [23,24]. Many somatic plants have been obtained from *Pinus* species, such as *P. pinea* [25], *P. thunbergii* [21,23,24,26], *P. radiata* [27], and *P. elliottii* [28]. However, the resistance of somatic plants to PWN has rarely been described.

RNA-seq is widely used to identify the different expression genes (DEGs) involved in plant and pathogen interactions [29,30]. Over the last decade, it was commonly used to elucidate the resistance mechanisms of pine to PWD. For example, in the study of *P. densiflora*, the phenylpropanoid biosynthesis, flavonoid biosynthesis, and oxidation–reduction pathways were found to have significant enrichment in response to *B. xylophilus* infection [31]. Modesto et al. [32] considered cell wall reinforcement and hormone signaling mechanisms essential for the resistance of *P. pinaster* to *B. xylophilus.* Wang et al. [33] found the oxidoreductase activity pathway was rapidly activated in response to invasion at 24 h of PWN infestation of *P. thunbergii*. However, the mechanisms behind the successful defense against PWN in *P. thunbergii* remain unknown. Previously, we compared changes in DEGs during disease development in resistant and susceptible *P. thunbergii* (i.e., (ph_1d vs. ph_3d) vs. (kh_1d vs. kh_3d)). The importance of α-linolenic acid metabolism and linoleic acid metabolism in nematode-resistance was found [34]. In this article, we highlighted and analyzed the DEGs in resistant and susceptible *P. thunbergii* at the same time point (i.e., ph_1d vs. kh_1d) during disease development.

A comparative transcriptome analysis (RNA-seq) of the shoot tissues of resistant and susceptible *P. thunbergii* following PWN inoculation was carried out to describe the transcriptional differences between the two groups. We used RNA-seq to identify differentially expressed genes (DEGs) between resistant and susceptible *P. thunbergii* at various time periods after inoculation. The identification of genetic determinants for PWD resistance is crucial for the PWD-resistant clones of *P. thunbergii*, as well as providing valuable data for resistance gene screening and breeding tactics in other *Pinus* species.

## 2. Results

### 2.1. Resistance Performance of P. thunbergii Somatic Plants

**Figure 1:**
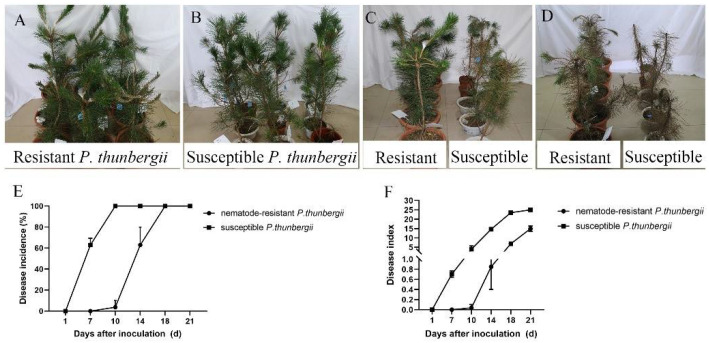
Performance of resistant and susceptible *P. thunbergii* after inoculation with pine wood nematode. Data represent mean ± SD. The performance of resistant and susceptible *P. thunbergii* infected with pine wood nematode for one (**A**,**B**), two (**C**), and four weeks (**D**). (**E**,**F**) indicate disease incidence and disease index of pine trees.

The somatic plants of nematode-resistant *P. thunbergii* exhibited disease symptoms about two weeks later than the susceptible *P. thunbergii* seedlings. After 7 days post-infestation (dpi), the tips of the upper branches at the inoculated sites of susceptible *P. thunbergii* began to droop, and a few needles near the inoculated sites started to lose their luster and even showed a loss of green phenotype, while the resistant *P. thunbergii* remained healthy (Figure 1A,B). After 14 dpi, the susceptible *P. thunbergii* showed obvious PWD symptoms, while the nematode-resistant *P. thunbergii* just began to develop the disease (Figure 1C), exhibiting distinct PWD characteristics only after 28 dpi (Figure 1D). Further statistical research revealed that after 7 dpi, the disease incidence of susceptible *P. thunbergii* was 66.67%, and the disease index was 0.70, whereas resistant *P. thunbergii* was 0. At this time point, the resistant *P. thunbergii* were healthy, which indicated that they suppressed PWN infestation. However, after 14 dpi, the disease incidence and index of the resistant *P. thunbergii* were up to 62.96% and 0.85, respectively, while for the susceptible *P. thunbergii* they were 100% and 14.67, respectively (Figure 1E,F). Our results showed that somatic plants were effective in delaying the onset of the PWD. Although the somatic plants finally developed PWD symptoms, it was two weeks later than the susceptible *P. thunbergii*. This development provided potential data to further elucidate PWD pathogenesis and resistance mechanisms.

### 2.2. Transcriptome Assemblies of Resistant and Susceptible P. thunbergii

To understand the molecular basis of *P. thunbergii* responses to PWN, we analyzed the temporal progression of the pathogenic invasion of the hosts. A comparative transcriptomic analysis (RNA-seq) was conducted in the shoot tissues of resistant and susceptible *P. thunbergii* following PWN inoculation. The DEGs were selected by comparing infected resistant trees with susceptible ones at fixed times. Seven cDNA libraries created from samples taken at four time points (a total of 239.14 Gb of clean data) were obtained by the transcriptome analysis of 21 samples. The clean data of each sample were >10.33 Gb, and the percentage of the Q30 base was >93.90% (Table S2). Our results revealed that the number of unigenes obtained was 120,249, and the number of transcripts was 192,025, while the average length of N50 unigenes was 1551 bp. BUSCO was employed to evaluate the integrity of the assembly, wherein the scores of unigenes and transcripts were 76.7% and 85%, respectively (Table S3).

### 2.3. Transcriptomic Responses to PWN Inoculation of P. thunbergii

**Figure 2:**
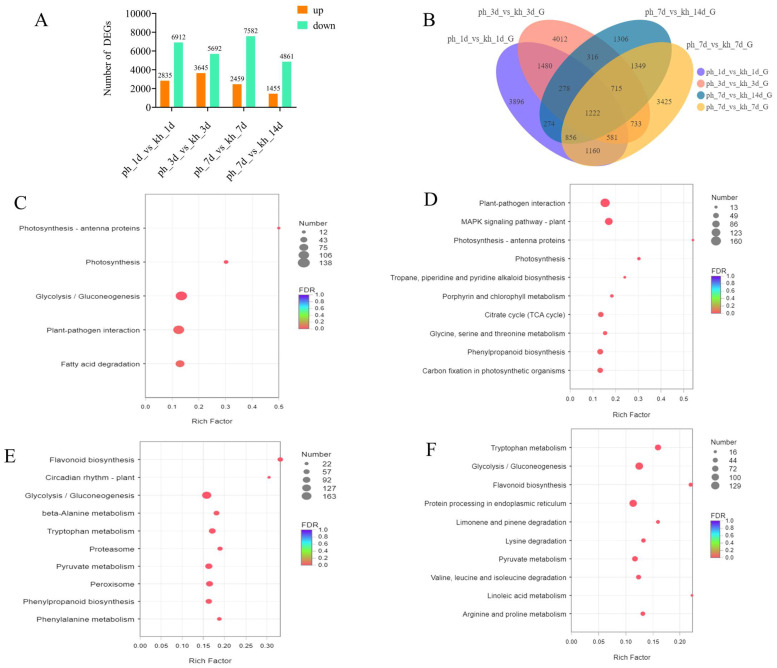
Comparisons and analysis of DEGs between resistant and susceptible *P. thunbergii* at four time points. (**A**) number of DEGs obtained in resistant and susceptible *P. thunbergii* at 1, 3, 7, and 14 dpi. (**B**) Venn diagram depicting the number and overlapping relationships of DEGs between different phenotypes at four time points. (**C**–**F**) indicate the KEGG enrichment analysis (*p* < 0.05) for resistant and susceptible *P. thunbergii* at 1, 3, 7, and 14 dpi, respectively. Resulting *p*-values were adjusted for control of the false discovery rate (FDR). Circle color denotes the FDR, circle size is proportional to the number of genes involved in the enrichment of the pathway (Count). ph indicates susceptible *P. thunbergii*; kh indicates nematode-resistant *P. thunbergii*.

To explore the global differences in the defense responses of susceptible and resistant *P. thunbergii* inoculated with PWN, significant DEGs were applied for further analyses at four time points. The result revealed that the maximum DEGs occurred at 7 dpi (total NO. 10,041) when the resistant *P. thunbergii* trees were healthy and susceptible *P. thunbergii* began to exhibit disease symptoms. The lowest number of DEGs occurred at 14 dpi (total No. 6316) when both resistant and susceptible *P. thunbergii* began to show disease symptoms (needles lost their green color). At 3 dpi, the number of up-DEGs was the highest of the four time points, which could be critical for *P. thunbergii* resistance to PWN inoculation. Furthermore, we discovered that the number of down-DEGs was consistently higher than the number of up-DEGs at each time point, implying that more DEGs were depressed during PWN inoculation in resistant *P. thunbergii* than in susceptible *P. thunbergii*, with the highest number of down-DEGs occurring at 7 dpi (No. 7582) (Figure 2A).

At different time points, there were large numbers of common DEGs. For example, 3561 common DEGs were found between 1 and 3 dpi, while 1222 common DEGs existed in four time points. In addition, several specific DEGs were present at four time points of PWN inoculation. For example, there were 3896, 4012, 3425, and 1306 special DEGs found at 1, 3, 7, and 14 dpi, which indicated that the defense responses of resistant and susceptible *P. thunbergii* might share some of the same basic defense responses, as well as vary with the disease progression (Figure 2B). To further elucidate the responses of resistant and susceptible *P. thunbergii* to PWN, we performed a KEGG enrichment analysis of these DEGs at four time points. Approximately 27.77% of the DEGs (numbering 33,367) were annotated by the KEGG database (Table S4). We evaluated the pathway at four time periods with a significance level of *p* < 0.05. Five significantly enriched pathways linked with the DEGs were discovered at 1 dpi, and pathway enrichment analyses using KEGG terms revealed a considerably larger number of metabolic pathways enriched in subsequent responses at 3, 7, and 14 dpi compared to 1 dpi. This indicated that *P. thunbergii* activated additional response mechanisms following PWN inoculation at 1 dpi. Photosynthesis antenna proteins and photosynthesis processes ranked among the most significantly enriched at 1 and 3 dpi (Figure 2C,D), while flavonoid biosynthesis ranked among the most significantly enriched at 7 and 14 dpi (Figure 2E,F). In addition, circadian rhythm-plant and linoleic acid metabolism showed higher enrichment levels at 7 and 14 dpi, respectively (Figure 2). Plant immunity pathways, i.e., plant–pathogen interactions and plant MAPK signaling pathways, increased significantly (*p* < 0.001) at 3 dpi. Phenylpropanoid biosynthesis was stimulated significantly (*p* < 0.05) at 3 and 7 dpi. Furthermore, at 7 and 14 dpi, the main enriched pathways involved amino acid metabolism (e.g., tryptophan, phenylalanine, and pyruvate metabolism) (Figure 2; Table S5). This could have been related to the creation of downstream secondary metabolites that aid in PWN defense. Thus, PWN infection caused complex and diverse response mechanisms involved in both growth and defense, including photosynthesis, glycolysis/gluconeogenesis, amino acid metabolism, and secondary metabolite biosynthesis.

### 2.4. Photosynthesis-Related DEGs Responses to PWN Inoculation in P. thunbergii

Photosynthesis and photosynthesis–antenna protein pathways were significantly enriched (*p* < 0.001) at 1 and 3 dpi, which prompted us to investigate the differences in gene-regulated growth between PWN-resistant and susceptible *P. thunbergii*. Subsequently, the DEGs associated with photosynthesis were examined. Interestingly, at 1 dpi, the 23 (100%) DEGs for photosynthesis and 12 (100%) DEGs for photosynthesis–antenna protein were upregulated in resistant *P. thunbergii*. At 3 dpi, all genes expressed upregulation except for one DEG (*HLCB1*, TRINITY_DN84,366_c0_g1), which was downregulated and involved the photosynthesis–antenna protein pathway in PWN-resistant *P. thunbergii* (Tables S6 and S7). Nematode-resistant *P. thunbergii* had considerably more DEGs encoding photosystem I, photosystem II, the cytochrome b6/f complex, photosynthetic electron transport, and F-type ATPase than susceptible *P. thunbergii*. This finding indicated that the photosynthetic system pathway was considerably impacted and may play a role in *P. thunbergii* responses to PWN inoculation.

### 2.5. Gene Regulation Involved in Pattern-Triggered Immunity and Effector-Triggered Immunity in PWN-Induced Immunity of P. thunbergii

**Figure 3:**
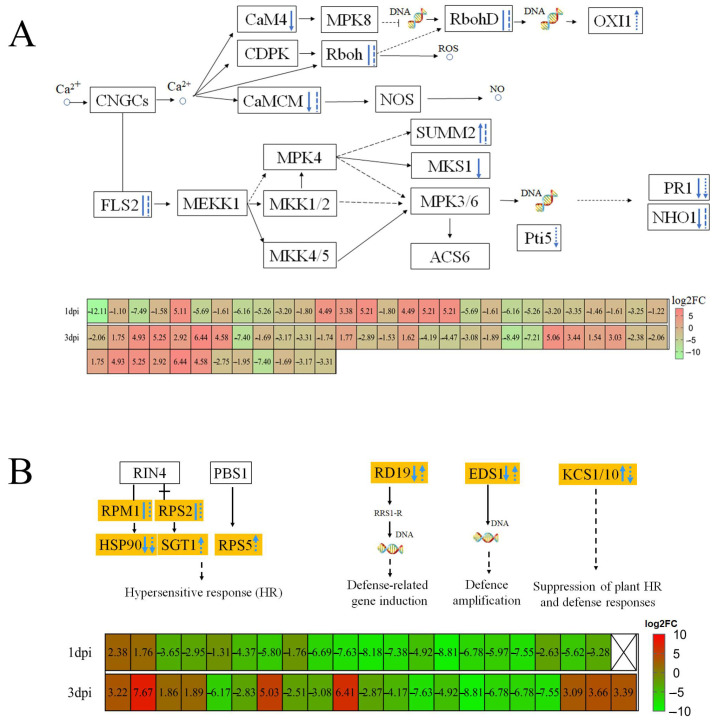
Transcriptome analysis revealed that a pine wood nematode infestation elicited pattern-triggered immunity and effector-triggered immunity defense responses. (**A**) Calcium ion-triggered immune response. (**B**) Resistance gene-induced immune responses. Solid lines represent 1 dpi, dashed lines represent 3 dpi, downward arrows represent lower DEG expression in resistant *P. thunbergii* than susceptible *P. thunbergii* and vice versa with the upward arrows, and straight and dashed lines represent both up- and downregulated DEGs. Solid and dashed arrows indicate characterized and predicted pathway steps, respectively.

We investigated the DEGs connected to *P. thunbergii*’s defense responses based on the gene expression profile and KEGG enrichment of the organism inoculated with PWN. This study found that after PWN infection, calcium ion-induced reactive oxygen species (ROS); NO signaling pathways; DEGs encoding *respiratory burst oxidase homologs (RBOH)*; and *calmodulin* (*CALM/CML*) were significantly altered. Additionally, the two trends were reversed. Nematode-resistant *P. thunbergii* exhibited higher expression levels in the ROS-regulated branch (three (75%) DEGs for RBOH at 1 dpi and six (85.71%) at 3 dpi) compared to susceptible *P. thunbergii*. But nematode-resistant *P. thunbergii* showed lower expression of ten (100%) DEGs of *calmodulin* (*CALM*/*CML*) at 1 dpi and seven (87.50%) at 3 dpi in the NO-regulated branch compared to susceptible *P. thunbergii* (Figure S1). *Calmodulin* expression was inhibited in nematode-resistant *P. thunbergii*; nevertheless, calcium ion control of ROS was elevated, which may have contributed to ROS formation. This finding was supported by the overexpression of *oxidative stress-inducible 1* (*OXI1*), which maintained ROS homeostasis at 3 dpi (Figure 3A). Next, we looked at the hypersensitive response (HR) and defense-related genes involved in pattern-triggered immunity (PTI) and effector-triggered immunity (ETI). For example, *SUMM2* (*disease resistance protein RPS2*) expression was greater in nematode-resistant *P. thunbergii* at 1 dpi but decreased at 3 dpi, whereas *MAP kinase substrate 1* (*MKS1*) expression was repressed.

In early PWN inoculation (at 1 dpi and 3 dpi), the expressions of *heat shock protein 90* (*HSP90*), *pathogenesis-related 1*(*PR1*), and *NHO1* (*glycerol kinase*) were suppressed in nematode-resistant *P. thunbergii*. The defense-related genes *RD19* (*cathepsin F*) and *EDS1* (*enhanced disease susceptibility 1 protein*) were downregulated at 1 dpi and then upregulated at 3 dpi; however, the *KCS* (*3-ketoacyl-CoA synthase*) expression showed an opposite trend (Figure 3B). It was found that the HR-gene *disease resistance protein RPS5* (*RPS5*) and *SGT1* (*suppressor of G2 allele of SKP1*), as well as the defense-related genes *RD19* and *EDS1*, were upregulated. Furthermore, the suppression of plant HR and defense response KCS genes was downregulated at 3 dpi, implying that pines’ immunity to PWN was significantly increased at 3 dpi after PWN infection. The defense of *P. thunbergii* against PWN was complex and required the regulation of multiple genes.

### 2.6. Signaling Molecule Responses to PWN Infection of P. thunbergii

**Figure 4:**
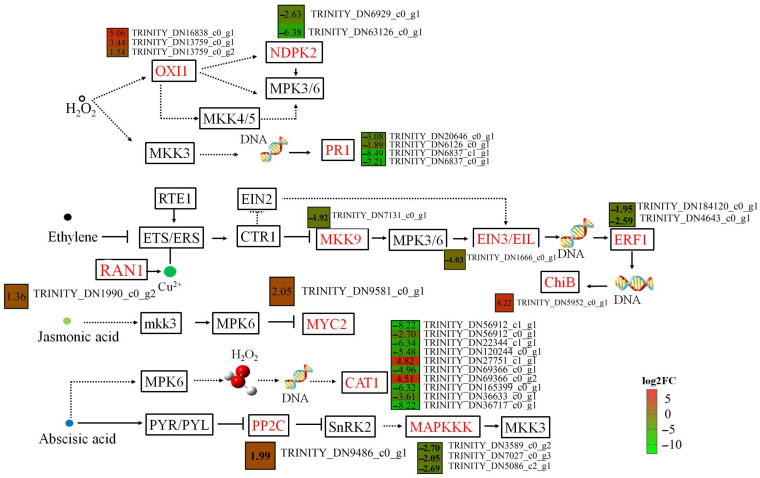
PWN infestation induced changes in response genes for phytohormones and reactive oxygen species. Solid and dashed arrows indicate characterized and predicted pathway steps, respectively. The red content in the box represented DEGs.

The ROS signaling system plays a key role in regulating plant stress responses. To further understand the physiological alterations involved in PWN infection, we investigated ROS scavenging in *P. thunbergii*. From transcriptome sequencing, a total of 13 DEGs related to ROS scavenging were identified at four time points (Table S8). Among these DEGs, the expression levels of *superoxide dismutase* (*SOD*) and *peroxidase* (*POD*) were higher in nematode-resistant than susceptible *P. thunbergii* at each time point, although *catalase* (*CAT*) expression was the opposite. Six upregulated DEGs encoding for *SOD* and *POD* were found only in the nematode-resistant *P. thunbergii*. Three of the four genes encoding for *glutathione reductase* were more highly expressed in susceptible *P. thunbergii*, while one was more highly expressed in nematode-resistant *P. thunbergii*. Furthermore, one encoding *phenylalanine ammonia lyase* (*PAL*) gene exhibited higher expression in susceptible *P. thunbergii* early in the PWN inoculation (~three times higher than in nematode-resistant *P. thunbergii*) (Table S8). These results implied that two completely different reactive oxygen species scavenging systems were activated when resistant and susceptible *P. thunbergii* was threatened by pine wood nematode. In the ROS-regulated branch, three DEGs encoding for *OXI1* were upregulated in PWN-resistant *P. thunbergii*, whereas two DEGs encoding for *NDPK2* were suppressed in PWN-resistant *P. thunbergii* (Figure 4). We speculated that the gene *OXI1* may have a negative regulatory effect on *NDPK2.*

In the ethylene signaling pathway, the expression of *Mitogen-Activated Protein Kinase Kinase Kinase 9* (*MKK9*), *ethylene-insensitive 3* (*EIN3*), and *Ethylene Response Factor 1* (*ERF1*) genes were downregulated, which suggested that the ethylene pathway was suppressed. This resulted in the up-expression of *Chitinase B* (*ChiB*) in nematode-resistant *P. thunbergii* at 3 dpi. Transcription factor *MYC2* (a core protein in the JA-induced signaling pathway), was up-expressed in nematode-resistant *P. thunbergii*. Furthermore, the *MKK3*-*MPK6*-*MYC2* module positively regulated abscisic acid (ABA) biosynthesis and signaling in *Arabidopsis* [35]. In this work, the positive regulator of ABA responses (*PYL*) was up-expressed, while an ABA signaling repressor (*protein phosphatase type 2C* (*PP2C*)) was down-expressed at 1 dpi. This suggested that the ABA pathway was activated; however, at 3 dpi, the *PYL* did not change, and the repressor *PP2C* was up-expressed, which resulted in *MAPKKK* (17-18) being repressed (Figure 4; Table S9). *MAPKKK17* was induced in response to a mite attack in *Arabidopsis* [36], while *MAPKKK18* (an ABA-activated kinase) was regulated by *PP2C*, which inhibited the kinase activity of *MAPKKK18* [37]. The reduced transcription of *MAPKKK18* in *Arabidopsis* exhibited obviously delayed leaf senescence [38]. Additionally, *CAT1* was indirectly regulated by ABA, with the expression of two of the ten genes encoding for *CAT1* being significantly upregulated and the other eight genes being downregulated. These results indicated several roles for the ABA pathway during early PWN infection.

**Figure 5:**
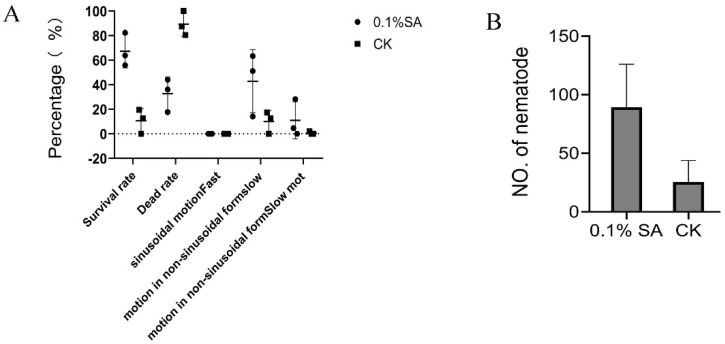
Effect of salicylic acid (SA) treatment on pine wood nematodes. (**A**) Pine wood nematode survival after SA treatments. (**B**) Proliferation of pine wood nematode following SA treatments. An amount of 0.1% SA represents the 0.1% (m/v) SA treatment (ethanol solvent), and CK represents the ethanol solvent control treatment. The data of (**B**) are expressed as the mean (±SE) of four replicates. Error bars represent the SE.

We studied the effect of salicylic acid (SA) on PWN survival and proliferation. The result showed that the PWN survival was higher when immersed in an alcohol solution containing 0.1% SA for 1h compared to the alcohol control solution (Figure 5A). Moreover, we found that a greater number of PWN were obtained in dishes sprayed with an alcohol solution containing 0.1% SA than in the control treatment sprayed with 10 mL of alcohol, for 3.5 times (Figure 5B). This indicates that SA could help PWN to resist stressful environments.

### 2.7. Flavonoid Biosynthesis Pathway with DEG-Related Interactions

**Figure 6:**
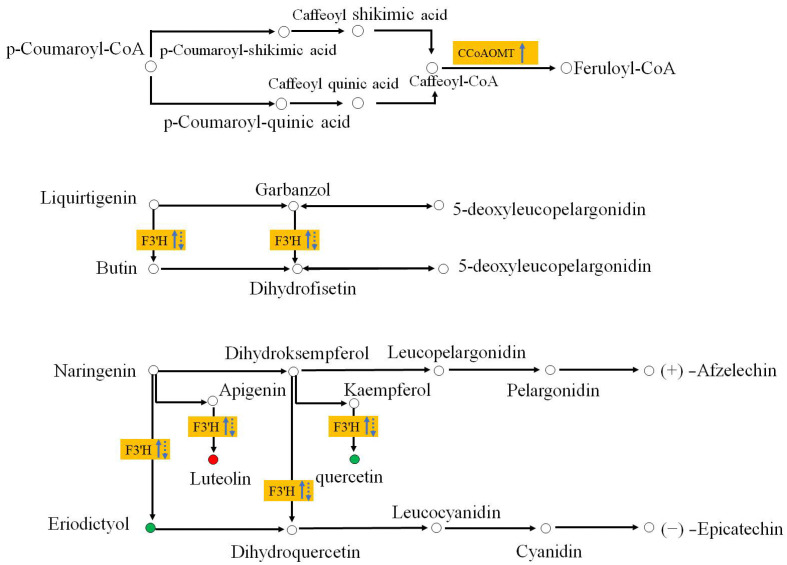
Flavonoid synthesis differences in PWN-inoculated *P. thunbergii*. Yellow boxes represent the DEGs between the resistant and susceptible *P. thunbergii*. Upward-pointing arrows represent up-expression, downward pointing arrows represent down-repression. Solid lines represent the transcription level at 7 dpi; dashed lines represent the transcription level 14 dpi. Red and green circles represent compounds detected in metabolomics, and red represents the abundance of compounds being higher in the resistant pine than the susceptible pine. Green indicates that the abundance of the compound was lower in resistant pine than the susceptible pine (at 7 dpi and 14 dpi).

We compared DEGs along the flavonoid biosynthetic pathway, significantly enriched (*p* < 0.001) at 7 and 14 dpi (Figure 6). Most enzyme activities were downregulated, except for *caffeoyl-CoA O-methyltransferase* (*CCoAOMT*) and *flavonoid 3′-monooxygenase* (*F3′H*), which were significantly upregulated (*p* < 0.05) at 7 dpi and downregulated at 14 dpi (Figures S3 and S4). Phenotypic results showed that nematode-resistant *P. thunbergii* did not exhibit disease symptoms at 7 dpi (upregulation of *CCoAOMT* and *F3′H*) but began showing needle chlorosis only at 14 dpi (*CCoAOMT* remained unchanged and *F3′H* was downregulated).

This result implied that *CCoAOMT* and *F3′H* modification might have been correlated with the delayed onset of disease for nematode-resistant *P. thunbergii*. The metabolomic results revealed that the luteolin metabolite catalyzed by *F3′H* was more abundant in resistant *P. thunbergii*, whereas the metabolites of eriodictyol and quercetin were more abundant in susceptible *P. thunbergii*. This observation raised interesting questions about the abundance of metabolites catalyzed by *F3′H*. The way in which *P. thunbergi* resisted late nematode infestation may be significantly impacted by the manner of control of these two metabolites, which was catalyzed by *F3′H*. Despite the lack of detection of *CCoAOMT* and *Feruloyl-CoA* in the metabolomics investigations, *CCoAOMT* has been linked to a number of leaf diseases (the maize *CCoAOMT* gene conferred resistance to both gray leaf spot and southern leaf blight, for example) [39]. These results revealed that *F3′H* and *CCoAOMT* were important components of the flavonoid metabolic response to protect against PWN. They may be useful for postponing the onset of disease in pine trees since this was especially the case in the early stages of the disease’s development.

### 2.8. Validation of RNA-seq Expression Data by qRT-PCR

**Figure 7:**
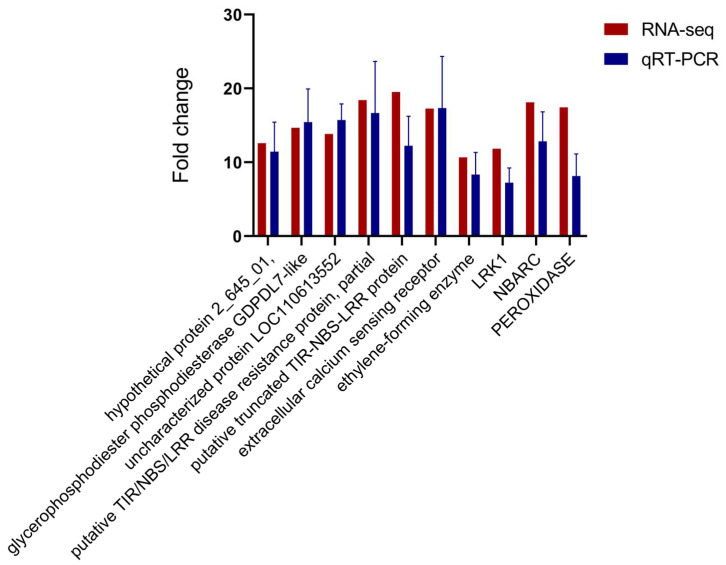
qRT-PCR validation of unigenes associated with resistance to PWN.

To validate the reliability of the RNA-seq results, 10 unigenes from the highly expressed DEGs were selected for qRT-PCR analysis. Expression of these unigenes differed significantly between susceptible and PWN-resistant *P. thunbergii* after infection with PWN (Figure 7). The unigenes selected for qRT-PCR analysis were those primarily involved in ROS-responsive genes, disease resistance protein, signal transduction, and integral components of the membrane. The expression pattern of selected unigenes indicated by qRT-PCR agreed well with RNA-seq.

Relative expression levels of qRT-PCR were calculated using *Elongation factor 1-alpha* as the internal control. The data are expressed as the mean (±SE). Error bars represent the SE.

## 3. Discussion

One of the most important steps in applying somatic plants for afforestation was determining resistance. Understanding *P. thunbergii*’s resistance mechanisms to the pine wood nematode was essential for enhancing integrated approaches to managing the worm, such as breeding for resistance or creating novel diagnostic instruments based on targeted resistance markers. According to our findings, susceptible *P. thunbergii* was more vulnerable to infection (~two weeks) than the somatic plants of resistant *P. thunbergii* (Figure 1). The transcriptomes of resistant and susceptible *P. thunbergii* shoot tissues differed remarkably (Figure 2). Compared to earlier studies that used suppression subtractive hybridization, more DEGs were obtained between resistant and susceptible *P. thunbergii* in this investigation employing next-generation sequencing [40]. The highest level of increased gene expression was seen in the number of DEGs at 3 dpi (NO. 3645), which may represent a crucial threshold for *P. thunbergii* resistance to PWN. Furthermore, when susceptible *P. thunbergii* started to wilt, and resistant *P. thunbergii* was healthy, the quantity of DEGs at 7 dpi (NO. 10041) was highest in the four examined time points. The KEGG enrichment analysis of DEGs revealed that the resistant and susceptible phenotypes differed in important KEGG pathways, indicating that their activated defense mechanisms and genes against PWN infection were qualitatively different.

### 3.1. Potential Roles of Photosynthesis in Resistance to PWN in Pine

In this investigation, we discovered that the photosynthesis–antenna proteins and photosynthesis pathways were considerably enriched (Figure 2C,D) and that several DEGs of PS I and PS II were upregulated in resistant *P. thunbergii* (Tables S6 and S7). Glycolysis and TCA cycle pathways were considerably changed (*p* < 0.001) at 1 and 3 days after infection. Plant photosynthesis produces sugars that travel via glycolysis and the TCA cycle, yielding intermediate metabolites, including pyruvate and acetyl coenzyme A. These compounds are gradually transformed into secondary metabolites, including terpenoids, phenols, and alkaloids, via a succession of oxidative folding and reduction processes. In contrast, pines use secondary metabolites as their principal defense against PWN [41,42]. We hypothesized that the high expression of these photosynthetic genes in resistant trees helped to inhibit PWN infestation and delay the development of PWD in resistant *P. thunbergii*. For host-pathogen interactions, photosynthetic regulation was developed as an approach to seek to correlate functional manipulation of PS I and PS II with host defense responses derived from primary metabolism [43]. After PWN infestation, photosynthesis-regulated genes responded first, followed by significant modifications in the circadian rhythm-plant pathway (*p* < 0.001). Our findings suggested that PWN infection disrupted plant growth, leading us to infer that wilting could be linked to altered growth.

The *light-harvesting chlorophyll protein complex* (*LHC*) was previously reported to be strongly phosphorylated in resistant wheat plants with stripe rust infection [44], while in this study, the DEGs related to *LHC* also showed a higher expression level in PWN-resistant *P. thunbergii* (Tables S6 and S7). Furthermore, the comparative transcriptomic analysis revealed that the majority of photosynthesis-related DEGs (photosystem I, II, cytochrome b6/f complex, photo-synthetic electron transport, and F-type ATPase) were expressed more in resistant *P. thunbergii* than in susceptible trees, implying an increase in photosynthetic efficacy. In a comparative study between poplar and *Lonsdalea populi*, higher expression levels of photosynthesis-related genes occurred in more highly resistant poplar, particularly for genes in the *LHC* [45]. Therefore, more robust photosynthesis might lead to higher levels of resistance in *P. thunbergii*, which might be an important regulator of PWN resistance. Generally, all these studies demonstrated the potentially critical role of photosynthesis in nematode resistance for *P. thunbergii*, which provided further new insights into the importance and engagement of photosynthesis-related genes in the regulation of PWN resistance.

### 3.2. Plant Recognition and Responses to PWN Invasion

In general, plants perceive changes in membrane surface transmembrane potential (Vm), Ca^2+^ influx, mitogen-activated protein kinase (MAPK) activation, and ROS burst caused by pathogen-associated molecular patterns (PAMP) [46]. These initial signaling events could further activate signal transduction pathways mediated by plant hormones, such as jasmonic acid (JA), salicylic acid (SA), and ethylene (ET) [47]. This eventually led to upregulated transcription levels of defense-related genes and increased levels of defense compounds [48]. The results of this study showed that PWN infection reduced the expression of *calmodulin* and changed that of *RBOH*, an important component of the ROS signaling network that affects a number of plant functions. *RBOH* was engaged in the production of H_2_O_2_ during *Arabidopsis thaliana* hypoxia stress [49], and it was also involved in the formation of aerenchyma in rice roots during oxygen-deficient conditions [50]. In an investigation of *Medicago truncatula* nodules, innate immunity was activated to mediate rhizobial infection and colonization, and *RBOH* and *calcium-dependent protein kinase* (*CDPK*) were important components of this process. A knock-out of *RBOH* (*MtRbohB* or *MtRbohD*) produced effective nodules [51]. Therefore, we hypothesized that calcium-triggered immunity during the early stage of PWN infection might inhibit infection by regulating ROS burst, stomata, and PWN colonization in host plants.

It has been observed that *NHO1* resistance to bacterial and fungal infections is dependent on non-host resistance [52,53]. Interestingly, we found that *NHO1* was significantly altered in *P. thunbergii* against PWN infection, indicating that it was involved in pine PWN resistance. The virulent bacteria appeared to suppress *NHO1*-mediated resistance by means of an Hrp-dependent virulence mechanism in *Arabidopsis thaliana* [52], which was consistent with the inhibition of *NHO1* at 1 dpi. Furthermore, *NHO1* compromised the resistance mediated by *RPS* and *RPM1*, and here, transcriptome analysis found that the expressions of *RPS* and *RPM1* were significantly altered, albeit whether they were regulated by *NHO1* requires further investigation. *OsNHO1* regulates rice resistance to bacterial blight and blast by influencing the wax content and regulating the transcription level of *PR* genes [54]. Here, we first noticed changes in *NHO1* resistance to PWN in *P. thunbergii*, however, its specific mechanism remains to be further explored.

The immediate increase in *heat shock proteins* (*HSPs*) was crucial for the cellular adaptation to environmental changes and for maintaining cellular homeostasis [55]. Prior transcriptome analysis of *P. thunbergii* found that *HSP70* expression was higher in resistant plants than in susceptible ones [40]. In contrast, our study found that *HSP90* expression was downregulated at both 1 and 3 dpi in the resistant phenotype. HSP70 and HSP90 may have various defense mechanisms in response to PWN infection. It was previously established that Mi-1-mediated nematode resistance in tomatoes needed the cooperation of *HSP90-1* [56]. *HSP90*, a highly conserved protein in most organisms and involved in numerous biological processes, may be implicated in plants’ fundamental responses to pathogenic organisms [57]. However, it is unclear how *HSPs* participate in the defense response to PWN infection. In summary, defense regulatory genes such as defense responsive (*PR1*, *NHO1*), hypersensitivity responsive (*Pti*, *Rboh*), and stress hormone responsive *ERF1* were suppressed in resistant *P. thunbergii* at 1 or 3 dpi, suggesting that resistant *P. thunbergii* has a protectively modulated defense mechanism during PWD. This was similar to the findings on tomato resistance to bacterial leaf spot (BLS) disease [58]. These findings contributed to a better knowledge of the pine response to PWN and will be essential tools for future PWN engineering.

Phytohormones SA, JA, and ET were signaling chemicals that played important roles in plant development and stress responses [59]. *MKS1* was necessary for full SA-dependent resistance, and overexpressing *MKS1* in *Arabidopsis thaliana* activated SA-dependent resistance [60]. *EDS1* increased SA production and accumulation while repressing the JA pathway, which was critical in the activation of effector-triggered immunity and plant basal defense [61,62,63], whereas *PR1* was a downstream response gene of SA.

In this way, the *EDS1*, *MKS1*, and *PR1* genes were down-expressed in resistant *P. thunbergii*, suggesting that the SA pathway was suppressed at 1 dpi. The action of SA on PWN was discovered to boost their survivability during alcohol stress and stimulate proliferation. Simultaneously, resistant *P. thunbergii* showed increased expression of *MYC2* (a key protein in the JA-induced signaling pathway). This demonstrated that suppressing SA and activating the JA pathway during early PWN infection could be beneficial for resistance. In a *P. pinaster* research, genes encoding JA biosynthesis enzymes (*LOX*, *OPRs*) and reacting to JA (*ERFs*, *MYC2*) were upregulated in a resistant genotype following inoculation. Additionally, the *EDS1* and SA had higher levels in the susceptible genotype, indicating that PWN resistance was associated with the activation of JA and suppression of the SA pathway [32], which aligned with the results of our study. JA-related regulatory gene up-expression also occurred in *P. massoniana* [64,65], *P. densiflora* [66], and *P. thunbergii* [40,67]. These results indicated that the JA pathway might have played a central role in response to PWN in several pine species. The suppression of SA and activation of the JA pathway during early PWN infection might be characteristic of an efficient resistant response in pines; however, further studies are needed.

*Ethylene response factors* (*ERFs*) are a large family of transcription factors that play a role in plant responses to biotic and abiotic stressors. Members of *ERFs* contributed to the integration of the JA and ET pathways. DEGs encoding *ERF1* were shown to be suppressed in resistant *P. thunbergii* at 3 dpi. *PYL* is a receptor that detects ABA and activates the ABA signaling pathway, which has been implicated in ABA-JA crosstalk during stress responses [68]. *PYL* was found to mediate the expression of *PP2C* and *MYC2*, which were involved in the ABA and JA signaling pathways, respectively [35]. In this work, the positive regulator of ABA response (*PYL*) was up-expressed and a repressor of ABA signaling, but *PP2C* was down-expressed at 1 dpi, suggesting activation of the ABA pathway. In *Arabidopsis thaliana*, the transcription factors *MYC* and *ERF* regulated the JA defensive response. ET was necessary to activate the *ERF* branch, whereas ABA inhibited this defense response and activated the *MYC* branch [69,70]. In this investigation, *P. thunbergii* appeared to have a similar regulatory mechanism, namely, the YPL up-expressed and activated the ABA signaling pathway. Furthermore, *ERF1* (a critical regulator of the ethylene route) was repressed, whereas *MYC2* (a key regulator of the JA pathway) was upregulated during early PWN infection. This seems to indicate a role for the ABA pathway in the early PWN infection of *P. thunbergii*. The immune regulation involved in hormones such as SA, JA, ET, and ABA appeared to be key to the differences in resistance to PWN in *Pinus* species. However, the extent to which numerous related genes were involved (e.g., *PYL*, *MKS1*, *EDS1*, *MYC2*, etc.) in contributing to PWN resistance still requires further validation. In addition, *MYC2* was a positive regulator of JAs-mediated flavonoid biosynthesis [71]. The *MYC2* was significantly upregulated, which may have led to differences in downstream flavonoid metabolism.

Following nematode recognition, the ROS produced by plants served as signaling molecules to activate defense responses, enhanced plant cell walls via crosslinking, and inhibited nematode migration [72]. However, higher ROS levels were toxic to plant cells, which might lead to their death if not removed in time [65]. In an earlier study of *P. thunbergii*, DEGs encoding for antioxidant enzymes were highly expressed in the resistance phenotype. For example, *POD* and *CAT* were more highly expressed in resistant *P. thunbergii* [40,68]. Similarly, the ROS-related genes were differentially expressed significantly between resistant and susceptible *P. thunbergii* when the branches of *P. thunbergii* were cut and placed in water for incubation and then inoculated with PWN [33]. In the current study, the expression levels of iron superoxide dismutase and peroxidase were higher in the resistant *P. thunbergii* than in the susceptible *P. thunbergii* at every time point. The greater expression of *SOD* was also found in pines with a higher resistance against PWN (e.g., *P. densiflora* [67], *P. pinaster* [32,73], *P. strobus* [74], *P. massoniana* [65], and *P. yunnanensis* [75]. Furthermore, *POD* contributed to cell wall synthesis by catalyzing the oxidative cross-linking of structural cell wall proteins (*extensin* and *HRGPs*), which were involved in cell wall-mediated resistance and played an important role in preventing pathogen intrusion [76,77]. As a result, the elevated levels of *POD* and *SOD* were advantageous in the protection against PWN.

### 3.3. Induction of Secondary Metabolic Pathways

**Figure 8:**
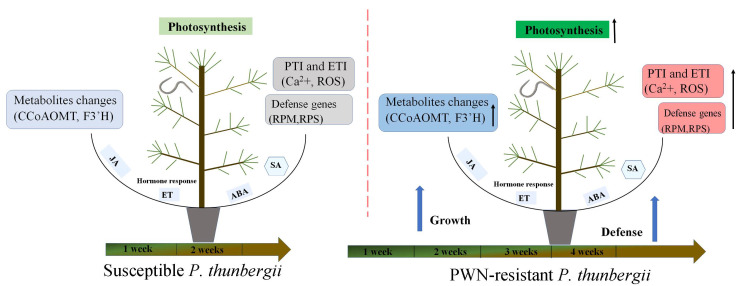
A proposed model of *P. thunbergii*’s defense response to *Bursaphelenchus xylophilus* infection during the onset period. Arrow up represents an upward trend.

Flavonoids are one of the most abundant types of phenolics, which are secondary metabolites produced by plants to protect themselves from diseases. Most enzymes in the flavonoid biosynthesis pathway were down-expressed at 7 and 14 dpi, but two important enzymes were dramatically up-expressed in the resistant genotype (Tables S3 and S4). One of these was *CCoAOMT*, which catalyzed the methylation of caffeoyl-CoA to produce feruloyl CoA [78]; *CCoAOMT* catalyzed the transfer of a methyl group from S-adenosylmethionine to a hydroxyl moiety of caffeoyl-CoA as part of the lignin biosynthetic pathway [79]. This enzyme played an important role in the lignin biosynthesis pathway, being involved in the phenylpropanoid pathway and lignin formation [80]. The suppression of *PrCCoAOMT* expression in *P. radiata* affected the lignin content and composition, resulting in a decrease in lignin content and an increase in the p-hydroxyphenyl/guaiacyl ratio relative to untransformed controls [81]. Furthermore, the *ZmCCoAOMT2* of maize exhibited gene conferring quantitative resistance to both southern leaf blight and gray leaf spot [39]. Lignin is a common polymer found in the cell walls of all vascular plants, hardening and strengthening cell wall structures via covalent crosslinking with cell wall polysaccharides [82]. Based on the positive regulatory link between *CCoAOMT* and lignin production, we predicted that up-expression of *CCoAOMT* at 7 dpi could induce PWN resistance, resulting in the cell wall reinforcement to restrict the further invasion by PWN. Another essential enzyme was *flavonoid 3′-monooxygenase* (*F3′H*, EC 1.14.14.82), which could catalyze multiple substrates in the flavonoid biosynthesis pathway [83]. Interestingly, our study revealed that the modification of *F3’H* resulted in the different accumulation of multiple metabolites. Metabolome results showed that eriodictyol and quercetin had lower levels in resistant plants at 7 dpi; however, luteoin was the opposite. Moreover, the concentration of luteoin was significantly higher in resistant *P. thunbergii* at each time point (Figure S2), which might be developed as a marker metabolite for assessing resistance to PWN in *P. thunbergii.* Based on the findings of this study, we summarized a proposed *P. thunbergii* defensive model against PWN infestation (Figure 8). This study provided valuable data on *P. thunbergii*’s resistance against PWN. These findings will undoubtedly help us better understand the transcriptional defense mechanisms against PWN in *P. thunbergii* and other *Pinus* species. However, more extensive functional research of the important genes identified in this study is needed in the future to untangle these complex defense systems.

*Bursaphelenchus xylophilus* infection triggered *P. thunbergii* PTI and ETI, phytohormone production, and induction of metabolites, which integrated multiple regulatory circuits. During the early stages of PWN infection, resistant *P. thunbergii* expressed more photosynthesis-related genes than susceptible *P. thunbergii*. Meanwhile, *RPS* and *RPM*, calcium-induced immune response genes, and ROS defense genes were all substantially expressed in resistant *P. thunbergii*. In the late stages of pine wood nematode infestation, secondary metabolism regulation took precedence, and resistant *P. thunbergii* had increased expression of secondary metabolism-regulating genes (*CCoAOMT* and *F3’H*).

## 4. Materials and Methods

### 4.1. Plant Materials and Nematode Infections

For this experiment, the PWN-resistant *P. thunbergii* somatic plants were used as resistant materials. The somatic plants were from the nematode-resistant *P. thunbergii* family 39 [24]. And somatic plants were the regeneration plants produced by somatic embryogenesis in nematode-resistant *P. thunbergii* [84]. The disease incidence of PWN-resistant *P. thunbergii* family 39 was lower by ~30–40% compared to susceptible *P. thunbergii* following artificial inoculation with the PWN (Wu et al., 2008). The PWN-resistant *P. thunbergii* family (No. 28–40) was introduced from Japan in 2004 [85,86]. The susceptible *P. thunbergii* (mortality rates were all 100% after inoculation with PWN in the previous test) from Suqian City, China, was selected as the control [34].

Three-year-old somatic plants and susceptible *P. thunbergii* were transferred to seedling pots for four months, and then the PWN-resistant and susceptible *P. thunbergii* with a similar growth status were selected for inoculation with the PWN. The PWN used in this experiment was the highly virulent *B. xylophilus* strain AMA3 isolate. Nematode infection was conducted as described earlier by our group [34]. The average heights of the resistant and susceptible trees were 1.47 m and 1.51 m, respectively. The branches growing at ~10 cm below the tips of the shoots were cut at an angle (~1 cm long × 1 mm deep) into the xylem. Subsequently, 1000 nematodes (suspended in 1 mL of sterile water) were injected into the longitudinal wounds of the resistant and susceptible *P. thunbergii* trees. Three branches per tree were inoculated (i.e., 3000 nematodes per pine tree). Samples were collected at 1, 3, 7, and 14 dpi to assess the resistance. Nine pine trees were used as replicates for each treatment. We sampled inoculated trees (both susceptible and resistant trees) at 1, 3, and 7 dpi; resistant trees were also sampled at 14 dpi. The last sampling time was chosen based on previous results, where the needles of resistant and susceptible *P. thunbergii* turned yellow at 14 and 7 dpi, respectively.

### 4.2. Assessment of PWN Resistance of Somatic Plants

The PWN resistance of *P. thunbergii* was divided into six grades: (0) no obvious symptoms; (1) a few needles lost their green color; (2) less than 1/3 needles lost their green color; (3) 1/3 to 1/2 needles lost their green color; (4) more than 1/2 needles lost their green color; and (5) all needles lost their green color). The disease grade and index were calculated based on symptoms over 14 days (onset period). The statistical susceptibility index and susceptibility rate were recorded according to a previously described method.

Disease incidence = Number of diseased pines/Total number of plants investigated × 100%; Disease index = ∑(Number of diseased pines in corresponding grade × Representative value)/Total number of pines investigated × The representative value of the most severe disease grade [45].

### 4.3. Sample Collection and RNA Extraction

Sampling time points were established, including 1, 3, 7, and 14 (dpi), to assess the resistance of the somatic plants against PWD. At 1, 3, 7, and 14 dpi, the 2 cm long segments of the stems below the inoculation sites were cut off and immediately put into liquid nitrogen. On 14 dpi, only the PWN-resistant *P. thunbergii* stem tissues were collected, as the susceptible *P. thunbergii* stems were completely wilted. The last sampling time was carried out according to previous results, where the needles of resistant and susceptible *P. thunbergii* turned yellow after 14 and 7 dpi, respectively. Three trees representing resistant and susceptible *P. thunbergii* were selected as biological replicates for each treatment. Next, these samples were stored at −80 °C for further RNA extraction.

The total RNA was extracted from each PWN inoculated tree sample at four time points using TRIzol^®^ Reagent (Plant RNA Purification Reagent for plant tissue) according to the instructions, while genomic DNA was removed using DNase I (TaKara, Nanjing, China). The RNA degradation and contamination were monitored using 1% agarose gels, whereas the integrity and purity of the total RNA quality were determined via a 2100 Bioanalyser (Agilent Technologies, Santa Clara, CA, USA) and quantified using an ND-2000 (NanoDrop Technologies, Wilmington, DE, USA). Only high-quality RNA samples (OD260/280 = 1.8~2.2, OD260/230 ≥ 2.0, RIN ≥ 7.9, 28S:18S ≥ 1.0) were utilized to develop a sequencing library.

### 4.4. Differential Expression Analysis and Functional Enrichment

To identify the DEGs (differential expression genes) between the resistant and susceptible *P. thunbergii* samples, the expression levels of each gene were calculated according to the transcripts per million reads (TPM) method. The DEGs with |log2 (foldchange)| ≥ 1 and *P*-adjust ≤ 0.05 were considered as differentially expressed genes. Furthermore, the databases GO (Gene Ontology, http://www.geneontology.org (accessed on 11 February 2022)) and KEGG (Kyoto Encyclopedia of Genes and Genomes, http://www.genome.jp/kegg/ (accessed on 14 February 2022)) were used to identify functional-enrichment analysis.

### 4.5. UPLC-MS/MS-Based Non-Targeted Metabolomics Analysis

Samples used for metabolomics analysis were consistent with transcriptomics analysis. The samples were collected at 1, 3, 7, and 14 dpi. Similarly, on 14 dpi, only the PWN-resistant *P. thunbergii* stem tissues were collected. The 2 cm segments of stems, located below the inoculation sites, were excised and promptly immersed in liquid nitrogen. Metabolite Extraction: 50 mg pine stem segment tissue was accurately weighed, and the metabolites were extracted using a 400 µL methanol/water (4:1, *v*/*v*) solution with 0.02 mg/mL L-2-chlorophenylalanin as internal standard. The sample was then re-solubilized with 100 µL solution (acetonitrile: water = 1:1) and extracted by low-temperature ultrasonication for 5 min (5 °C, 40 KHz), followed by centrifugation at 13,000× *g* and 4 °C for 10 min. The supernatant was transferred to sample vials for LC-MS/MS analysis.

Quality control sample: As a part of the system conditioning and quality control process, a pooled quality control sample (QC) was prepared by mixing equal volumes of all samples. The QC samples were disposed of and tested in the same manner as the analytic samples. This helped to represent the whole sample set, which would be injected at regular intervals (every 5–15 samples) in order to monitor the stability of the analysis.

UHPLC-MS/MS analysis: The LC-MS/MS analysis of the sample was conducted on a Thermo UHPLC-Q Exactive HF-X system equipped with an ACQUITY HSS T3 column (100 mm × 2.1 mm i.d., 1.8 μm; Waters Corporation, Milford, NH, USA) at Majorbio Bio-Pharm Technology Co. Ltd. (Shanghai, China). The mobile phases consisted of 0.1% formic acid in water: acetonitrile (95:5, *v*/*v*) (solvent A) and 0.1% formic acid in acetonitrile/isopropanol/water (47.5:47.5, *v/v*) (solvent B). The flow rate was 0.40 mL/min, and the column temperature was 40 °C. MS conditions: the mass spectrometric data were collected using a Thermo UHPLC-Q Exactive HF-X Mass Spectrometer equipped with an electrospray ionization (ESI) source operating in positive mode and negative mode. The optimal conditions were set as follows: source temperature at 425 °C; sheath gas flow rate at 50 arb; Aux gas flow rate at 13 arb; ion-spray voltage floating (ISVF) at −3500V in negative mode and 3500 V in positive mode, respectively; Normalized collision energy, 20-40-60V rolling for MS/MS. Full MS resolution was 60,000, and MS/MS resolution was 7500. Data acquisition was performed via the Data Dependent Acquisition (DDA) mode. The detection was carried out over a mass range of 70–1050 *m*/*z*.

Data analysis: The pretreatment of LC/MS raw data was performed by Progenesis QI version 2.0 (Waters Corporation, Milford, NH, USA) software, and a three-dimensional data matrix in CSV format was exported. The information in this three-dimensional matrix included: sample information, metabolite name, and mass spectral response intensity. Internal standard peaks, as well as any known false positive peaks (including noise, column bleed, and derivatized reagent peaks), were removed from the data matrix, deredundant, and peak pooled. At the same time, the metabolites were identified by searching databases, and the main databases were the HMDB (http://www.hmdb.ca/ (accessed on 10 March 2022)), Metlin (https://metlin.scripps.edu/ (accessed on 11 March 2022)). The data matrix obtained by searching the database was uploaded to the Majorbio cloud platform (https://cloud.majorbio.com (accessed on 11 March 2022)) for data analysis. First, the data matrix was pre-processed, as follows: At least 80% of the metabolic features detected in any set of samples were retained. After filtering, for specific samples with metabolite levels below the lower limit of quantification, the minimum metabolite value was estimated, and each metabolic signature was normalized to the sum. To reduce the errors caused by sample preparation and instrument instability, the response intensities of the sample mass spectrometry peaks were normalized using the sum normalization method to obtain the normalized data matrix. Meanwhile, the variables of QC samples with relative standard deviation (RSD) > 30% were excluded and log10 logarithmicized to obtain the final data matrix for subsequent analysis. Then, the R package “ropls”(Version 1.6.2) was used to perform principal component analysis (PCA) and orthogonal least partial squares discriminant analysis (OPLS-DA), and 7-cycle interactive validation evaluating the stability of the model. The metabolites with VIP > 1, *p* < 0.05 were determined as significantly different metabolites based on the Variable importance in the projection (VIP) obtained by the OPLS-DA model and the *p*-value generated by Student’s *t* test. Differential metabolites among the two groups were mapped into their biochemical pathways via metabolic enrichment and pathway analysis based on the KEGG database (http://www.genome.jp/kegg/ (accessed on 20 March 2022)). These metabolites could be classified according to the pathways they are involved in or the functions they perform. The principle was that the annotation analysis of a single metabolite develops into an annotation analysis of a group of metabolites. Python package “scipy.stats” (https://docs.scipy.org/doc/scipy/ (accessed on 20 March 2022)) was used to perform enrichment analysis to obtain the most relevant biological pathways for experimental treatments.

### 4.6. Quantitative RT-PCR Analysis

The RNA samples used for the qRT-PCR and transcriptome sequencing were identical. To evaluate the accuracy and reproducibility of the RNA-sequenced (RNA-Seq) expression profiles, quantitative real-time polymerase chain reaction (qRT-PCR) analysis was performed to analyze the expression levels of regulated genes at four distinct time points. The primer pairs (Table S1) for the candidate genes were designed on the NCBI website. Quantitative RT-PCR was run in a 7900 Real-Time PCR System (Applied Biosystems, Foster City, CA, USA) with the SYBR Green detection method to verify the transcriptome sequencing results. Quantitative real-time PCR (qRT-PCR) was performed in a 20 μL reaction volume containing 10 μL of SYBR Green Master Mix (Vazyme Biotech, Nanjing, China).

### 4.7. Nematode Motility and Proliferation in Salicylic Acid Treatment

Salicylic acid (SA) solution: 0.01 g of salicylic acid was dissolved in 10 mL of ethanol solution (5 mL of ethanol and 5 mL of sterile water) at a concentration of 0.1% (*w*/*v*) of salicylic acid. SA treatments were in two ways: (1) 100 µL of suspension containing approximately 1000 nematodes was dropped into 10 mL of SA solution and shaken well, left for 30 min, and the movement of nematodes was observed with a light microscope. A 10 mL ethanol solution (5 mL of ethanol and 5 mL of sterile water) was used as a control. (2) 100 µL of suspension containing approximately 1000 nematodes was added onto potato dextrose agar (PDA) covered with *B. cinerea* and sprayed with 1mL of SA (0.1%) solution. And then, the nematodes were cultured in the dark at 28 °C for 7 days. The nematodes were collected using the Baermann funnel method for approximately 12 h and counted under a light microscope. The same number of nematodes was incubated on PDA plates and sprayed 1 mL of ethanol solution (ethanol/sterile water = 1:1 (*v*:*v*)) as a control group.

### 4.8. Statistical Analysis

Results were expressed as percentages using nonparametric methods. The data about gene expression trends after treatments were analyzed according to the methods of Livak and Schmittgen [87], using a one-way analysis of variance followed by Tukey’s honest significant difference (HSD) post hoc test. The analysis of the Venn diagram and heatmap was performed using R version 3.6.2. In addition, the graphs were created using GraphPad Prism (Patterns & Practices., Redmond, WA, USA) and Adobe Photoshop CS6 (64-bit) software (Adobe, San Jose, CA, USA). Functional analysis was performed using GraphPad Prism (Patterns & Practices, Redmond, WA, USA).

## 5. Conclusions

Under PWN stress, most of the DEGs were downregulated, which potentially led to the physiologic disorders of *P. thunbergii*. We systematically analyzed DEGs that were potentially related to PWN resistance. Our results demonstrated that photosynthesis, calcium-triggered immunity, and resistance phytohormone were rapidly induced to respond to the PWN infection (at 1 and 3 dpi). Furthermore, the increased activity of ROS-scavenging enzymes was a beneficial reaction to limit the rapid progression of disease in *P. thunbergii*. In the early stage of disease development (at 7 dpi), the metabolism of flavonoids might be important in influencing the delayed onset of disease in *P. thunbergii*. PWN-resistant *P. thunbergii* exhibited higher antioxidant enzyme activities, photosynthesis capacities, and JA pathway mediation. Overall, we revealed that nematode-resistant and susceptible *P. thunbergii* had different defense mechanisms in response to PWN. These results provide an important basis for the application of PWN-resistant plants in future artificial afforestation.

## Data Availability

The data described in this study can be found in the article and the Supplementary Materials.

## Supplementary Materials

The following supporting information can be downloaded at https://www.mdpi.com/article/10.3390/ijms25105156/s1.
