# Supplementary material for: Pinus thunbergii Parl. Somatic Plants’ Resistance to Bursaphelenchus xylophilus Depends on Pathogen-Induced Differential Transcriptomic Responses
Source: Int J Mol Sci. 2024 May 9;25(10):5156. doi: 10.3390/ijms25105156 (PMC11121521; doi:10.3390/ijms25105156)
Supplement: Supplementary file 1 [file ijms-25-05156-s001.zip › ijms-2963880-supplementary.pdf]

Table S1. Primer pairs used in this study. Elongation factor 1-alpha was used as the internal control.

Gene ID	Primers	
ethylene-forming enzyme	F: ACGAGAAGCAATACTATGAT	R: AATCTTTCCACACCACTT
hypothetical protein	F: TGGAGTGACCTCGTAGAA	R: ATGTGACTTGAATGAAGACTT
peroxidase 43	F: GAAGAGATGGAATGAGAT	R: CATTGAGAACAACAAGAT
TRINITY_DN9232_c0_g3 extracellular calcium sensing receptor	F: TTACCTATGCTTCTATCCA	R: GTATGTATCCTCCACCTT
TRINITY_DN7181_c0_g2 putative truncated TIR-NBS-LRR protein	F: ACGGTTATTCTGTGTTCT	R: CGAAGACTGTGGTAGATAT
TRINITY_DN2086_c1_g1 putative TIR/NBS/LRR disease resistance protein, partial	F: CTATACCTAATTGCTGCTCCAA	R: ACATATCATCGCCGTTCAC
TRINITY_DN2007_c0_g5 uncharacterized protein LOC110613552	F: TAGTTATGCCAATCTGATATG	R: ATCCTCTTCACCATCTTC
TRINITY_DN7835_c0_g2 glycerophosphodiester phosphodiesterase GDPDL7-like	F: TTACAAGCACCTACAAGT	R: GTCCACGAACTGAAGATA
LRK1	F: ATTTGGTGGTTGTCTTCT	R: CTTGATGTCCCTGTGAAT
NB-ARC	F: GAGAGGCGTGAGTATTGG	R: AACAGGCGACATCTAAGAAT
Elongation factor-1 alpha	F: AGATTGGAAATGGTTATGCCCCTGT	R: CCAGAACGCCTGTCAACCTTGGT

Table S2 Summary of resistant and susceptible RNA-seq reads with no reference genome

Sample	Clean reads	Clean bases	Q20 (%)	Q30 (%)	GC content (%)	Mapped reads	Mapped ratio
kh_14_3	71414328	1.06E+10	97.94	93.90	45.06	28250238	79.12%
kh_14_2	72342340	1.07E+10	98.27	94.69	45.62	29359458	81.17%
kh_14_1	70025020	1.03E+10	98.1	94.26	45.77	28384603	81.07%
kh_7d_3	79528446	1.18E+10	98.15	94.37	45.34	31796046	79.96%
kh_7d_2	72368066	1.07E+10	98.18	94.44	44.89	29624548	81.87%
kh_7d_1	72512164	1.07E+10	98.1	94.22	45.14	28805049	79.45%
kh_3d_3	83960312	1.24E+10	98.27	94.64	45.01	34238599	81.56%
kh_3d_2	78979550	1.17E+10	98.21	94.55	45.27	32535079	82.39%
kh_3d_1	73412854	1.08E+10	98.18	94.46	45.05	29953646	81.60%
kh_1d_3	72568534	1.07E+10	98.09	94.23	45.09	29653618	81.73%
kh_1d_2	86417954	1.28E+10	98.15	94.41	45.23	35098819	81.23%
kh_1d_1	72042226	1.07E+10	98.25	94.61	45.09	29513783	81.93%
ph_7d_3	72686716	1.07E+10	98.1	94.29	45.49	28505050	78.43%
ph_7d_2	72891214	1.08E+10	98.11	94.22	45.11	28055614	76.98%
ph_7d_1	74033248	1.09E+10	98.15	94.36	46.2	29452803	79.57%
ph_3d_3	85126248	1.26E+10	98.13	94.31	45.3	33817910	79.45%
ph_3d_2	79015534	1.17E+10	98.16	94.4	45.2	31255360	79.11%
ph_3d_1	84645650	1.25E+10	98.29	94.73	45.42	33763755	79.78%
ph_1d_3	74255326	1.09E+10	98.23	94.55	45.31	29942197	80.65%
ph_1d_2	91131434	1.35E+10	98.24	94.59	45.2	36241647	79.54%
ph_1d_1	78379906	1.16E+10	98.27	94.67	45.33	31036405	79.19%

Note: Clean reads: The calculation method is the same as raw reads, and the statistical files are filtered sequencing data. Clean bases: the number of clean reads was multiplied by the length to obtain the total base number after quality control. Q20 and Q30: the percentage of the number of bases with Phred value greater than 20 and 30 in the total base was calculated respectively (clean data). GC content (%): Percentage of the total number of G and C bases in the total number of bases (clean data). Mapped reads: the number of clean reads that can be mapped into assembled transcripts. Mapped ratio: percentage of clean reads that can be mapped into assembled transcripts.

Table S3. Assembly result evaluation

Type	Unigene	Transcript
Total number	120249	192025
Total base	124083040	211759866
Largest length (bp)	20577	20577
Smallest length (bp)	201	201
Average length (bp)	1031.88	1102.77
N50 length (bp)	1551	1707
E90N50 length (bp)	2941	2429
Fragment mapped percent (%)	61.781	79.117
GC percent (%)	43.67	42.93
TransRate score	0.28438	0.35782
BUSCO score	C:76.7% [S:69.9%; D:6.8%]	C:85.0% [S:53.3%; D:31.7%]

Note: N50: according to the length of the assembly of Unigene /transcript from large to small order, the length of the accumulated transcript to the total length of half, the length of the corresponding transcript. E90N50: for the expression in the top 90% of Unigene /transcript, according to the length from large to small order, the length of the accumulated transcript to the total length of half, the length of the corresponding transcript. Fragment mapped reads: the mapped rate obtained from all sample clean reads after merging and comparing with assembled Unigene /transcript. Busco score: Busco is used to evaluate the integrity of the assembly. The higher the score, the better the integrity (its specific meaning: "C" stands for complete, which represents the proportion of sequences reaching the desired length in the total sequences of BUSCO. It consists of two parts, in which "S" stands for single-copy, that is, a sequence can be aligned to a gene in the library; "D" means duplicate, in which a single sequence aligns multiple genes in the library).

Table S4. Number of annotated DEGs and percent

Database	Exp_Unigene number (percent)	Exp_Transcript number (percent)
GO	53605 (0.4461)	94321 (0.492)
KEGG	33367 (0.2777)	53568 (0.2794)
COG	58352 (0.4856)	96387 (0.5028)
NR	64373 (0.5357)	111899(0.5837)
Swiss-Prot	51377 (0.4275)	86108 (0.4492)
Pfam	56207 (0.4677)	94917 (0.4951)
Total_anno	73738 (0.6136)	124989 (0.652)
Total	120174 (1)	191704 (1)

Table S5 the KEGG enrichment analysis ($P < 0.05$) for resistant and susceptible *P. thunbergii* at 1, 3, 7 and 14 dpi.

Timepiont	Description	*P* value_corrected	First Category	Second Category
1 dpi	Photosynthesis - antenna proteins	3.24009E-05	Metabolism	Energy metabolism
	Photosynthesis	4.60408E-05	Metabolism	Energy metabolism
	Glycolysis / Gluconeogenesis	0.001195525	Metabolism	Carbohydrate metabolism
	Plant-pathogen interaction	0.035105343	Organismal Systems	Environmental adaptation
	Fatty acid degradation	0.045762632	Metabolism	Lipid metabolism
3 dpi	Plant-pathogen interaction	1.04E-09	Organismal Systems	Environmental adaptation
	MAPK signaling pathway - plant	5.09E-09	Environmental Information Processing	Signal transduction
	Photosynthesis - antenna proteins	1.10E-06	Metabolism	Energy metabolism
	Photosynthesis	4.54E-06	Metabolism	Energy metabolism
	Tropane, piperidine and pyridine alkaloid biosynthesis	0.008549288	Metabolism	Biosynthesis of other secondary metabolites
	Porphyrin and chlorophyll metabolism	0.010630604	Metabolism	Metabolism of cofactors and vitamins
	Citrate cycle (TCA cycle)	0.017482546	Metabolism	Carbohydrate metabolism
	Glycine, serine and threonine metabolism	0.018541753	Metabolism	Amino acid metabolism
	Phenylpropanoid biosynthesis	0.018888724	Metabolism	Biosynthesis of other secondary metabolites
	Carbon fixation in photosynthetic organisms	0.020513587	Metabolism	Energy metabolism
7 dpi	Flavonoid biosynthesis	1.46E-12	Metabolism	Biosynthesis of other secondary metabolites
	Circadian rhythm - plant	0.000430078	Organismal Systems	Environmental adaptation
	Glycolysis / Gluconeogenesis	0.000566535	Metabolism	Carbohydrate metabolism
	beta-Alanine metabolism	0.002106605	Metabolism	Metabolism of other amino acids
	Tryptophan metabolism	0.002131642	Metabolism	Amino acid metabolism
	Proteasome	0.002868328	Genetic Information Processing	Folding, sorting and degradation
	Pyruvate metabolism	0.003100707	Metabolism	Carbohydrate metabolism
	Peroxisome	0.003461039	Cellular Processes	Transport and catabolism
	Phenylpropanoid biosynthesis	0.009972333	Metabolism	Biosynthesis of other secondary metabolites
	Phenylalanine metabolism	0.010628951	Metabolism	Amino acid metabolism
14 dpi	Tryptophan metabolism	4.30E-10	Metabolism	Amino acid metabolism
	Glycolysis / Gluconeogenesis	1.03E-08	Metabolism	Carbohydrate metabolism
	Flavonoid biosynthesis	1.44E-08	Metabolism	Biosynthesis of other secondary metabolites
	Protein processing in endoplasmic reticulum	7.47E-06	Genetic Information Processing	Folding, sorting and degradation
	Limonene and pinene degradation	0.000202869	Metabolism	Metabolism of terpenoids and polyketides
	Lysine degradation	0.000552078	Metabolism	Amino acid metabolism
	Pyruvate metabolism	0.000553833	Metabolism	Carbohydrate metabolism
	Valine, leucine and isoleucine degradation	0.000581018	Metabolism	Amino acid metabolism
	Linoleic acid metabolism	0.000582324	Metabolism	Lipid metabolism
	Arginine and proline metabolism	0.000591076	Metabolism	Amino acid metabolism

Table S6 DEGs in Photosynthesis-antenna protein pathway

DEG names			1dpi		3dpi	
			log2FC	*P* value	log2FC	*P* value
photosystem I	Hlca1	TRINITY_DN4730_c0_g2	1.519	6.25081E-05	1.519	2.02408E-08
	Hlca2	TRINITY_DN1973_c0_g1	2.49	3.99461E-06	3.114	1.9836E-11
	Hlca3	TRINITY_DN154062_c0_g1	1.541	0.000149905	1.858	5.18131E-07
	Hlca4	TRINITY_DN25043_c0_g1	1.844	9.04112E-11	2.225	4.05093E-13
photosystem II	Hlcb1	TRINITY_DN6873_c0_g1	1.917	1.00018E-08	3.046	1.06983E-05
		TRINITY_DN781_c0_g2	1.842	2.86423E-07	2.361	2.90037E-06
		TRINITY_DN84366_c0_g1	x	x	-2.799	0.019883918
		TRINITY_DN84366_c0_g2	1.341	0.007988466	1.345	0.003958261
	Hlcb2	TRINITY_DN39975_c1_g1	2.060	0.0006	1.259	0.046923928
		TRINITY_DN781_c0_g1	1.962	1.56313E-05	2.336	4.59636E-06
		TRINITY_DN21077_c0_g1	3.133	2.64814E-09	2.346	0.000196146
	Hlcb4	TRINITY_DN17219_c0_g1	1.658	0.00156	1.952	1.96089E-07
	Hlcb5	TRINITY_DN9148_c0_g1	2.334	0.00013	3.063	4.17221E-14

x indicates the DEG was not detected.

Table S7 DEGs in Photosynthesis pathway

DEGs in Photosynthesis pathway			1 dpi		3 dpi	
			log2FC	*P* value	log2FC	*P* value
photosystem II	PsbA	x	x	x	1.452563047	0.016809785
	PsbC	x	x	x	2.541027549	0.004711721
	PsbO	TRINITY_DN23356_c0_g1	1.376479364	0.001306769	1.69019196	2.02053E-05
	PsbP	TRINITY_DN27885_c0_g1	1.432851994	0.000868634	1.575536079	5.45692E-07
	PsbQ	TRINITY_DN5189_c0_g1	0.260476368	0.999946751	1.715121891	5.09819E-10
	PsbR	TRINITY_DN522_c0_g2	2.382321903	1.03712E-06	2.845772888	1.4304E-13
	PsbS	TRINITY_DN53830_c0_g1	1.393433863	0.045407619	x	x
		TRINITY_DN53830_c0_g2	3.204503447	0.00156764	6.581488406	3.13469E-08
	PsbW	TRINITY_DN6517_c0_g1	1.372782276	0.00039685	1.619437899	2.60662E-10
	Psb27	TRINITY_DN175502_c0_g1	2.582181198	3.58134E-05	3.355282065	3.39427E-21
	Psb28	TRINITY_DN129969_c0_g3	2.284046351	4.94231E-07	1.936193837	3.29654E-05
photosystem I	PsaD	TRINITY_DN47002_c0_g1	1.936568128	0.000127832	2.296589954	1.89951E-05
	PsaE	TRINITY_DN12818_c0_g1	1.442267263	0.01241275	1.686460729	7.05391E-06
	PsaF	TRINITY_DN1365_c0_g1	1.39422917	0.000418575	1.898663025	6.06659E-13
	PsaG	TRINITY_DN2381_c0_g1	1.621243125	0.00469906	1.897524498	1.78218E-05
	PsaH	TRINITY_DN7693_c0_g1	2.007699438	1.91622E-12	1.85731545	8.2582E-12
	PsaK	TRINITY_DN8356_c0_g1	2.172777898	0.000106442	2.566777345	1.36039E-12
	PsaL	TRINITY_DN9972_c0_g1	1.920745409	5.79025E-05	2.07863529	1.85763E-05
	PsaN	TRINITY_DN5240_c0_g1	1.217331616	0.033426744	0.709873338	0.784244094
	PsaO	TRINITY_DN78980_c0_g1	2.579778149	1.01953E-07	2.618903551	6.9753E-23
Cytohrome b6/f complex	PetC	TRINITY_DN68779_c0_g1	1.175665577	0.000282715	x	x
Photosynthethic electron transport	PetF	TRINITY_DN9953_c0_g1	2.365019226	2.02438E-10	2.76862624	3.2952E-06
	PetH	TRINITY_DN24944_c0_g2	1.733371839	0.000205218	2.195080929	1.15333E-08
F-type ATPase	gamma	TRINITY_DN3468_c0_g5	1.633483897	0.021380468	2.078692451	0.000139901
	delta	TRINITY_DN1564_c0_g1	1.587184401	0.016342082	1.978052588	3.27557E-06

x indicates the DEG was not detected.

Table S8 Expression of oxidoreductase genes

Gene name	Gene id	Resistant *P. thbergii*				Susceptible *P. thunbergii*		
		14dpi	7dpi	3dpi	1dpi	7dpi	3dpi	1dpi
superoxide dismutase	TRINITY_DN13143_c0_g1	8.183	6.073	6.967	7.607	1.867	1.683	3.017
	TRINITY_DN11861_c0_g2	0.703	4.500	12.277	12.387	1.720	1.863	3.080
peroxidase	TRINITY_DN24293_c0_g1	2.277	13.857	30.103	36.890	6.017	8.740	7.617
	TRINITY_DN21574_c1_g1	2.387	3.383	7.617	5.237	0.747	0.627	0.323
	TRINITY_DN4737_c0_g1	31.103	149.853	376.997	380.383	71.757	161.630	97.720
	TRINITY_DN15916_c0_g1	5.283	9.847	23.617	23.483	5.057	8.950	13.650
	TRINITY_DN18540_c0_g1	2.750	10.153	26.197	15.357	2.610	2.407	6.153
catalase	TRINITY_DN56912_c0_g1	0.310	0.007	0.130	0.000	3.153	0.730	1.027
	TRINITY_DN36633_c0_g1	0.100	0.097	0.063	0.147	1.253	0.640	0.527
glutathione reductase	TRINITY_DN23170_c0_g1	1.927	2.493	3.743	4.000	0.280	0.323	0.607
	TRINITY_DN37828_c0_g1	0.280	0.040	0.090	0.070	1.573	0.903	6.690
	TRINITY_DN12751_c0_g1	1.220	2.110	0.523	0.247	5.503	7.860	4.253
phenylalanine ammonia lyase	TRINITY_DN8407_c0_g2	4.787	8.507	3.800	3.893	6.787	11.903	11.950

Table S9. The DEGs involved in plant hormones and reactive oxygen species

Gene names	Seq ID	3dpi		1dpi	
		log2FC	*p* values	log2FC	*p* values
OXI1	TRINITY_DN16838_c0_g1	5.0647	0.0016	0.8887	0.6986
	TRINITY_DN13759_c0_g1	3.4363	0.0004	-0.1988	0.9563
	TRINITY_DN13759_c0_g2	1.5382	0.0212	-0.1896	0.8869
NDPK2	TRINITY_DN6929_c0_g1	-2.6311	0.0213	-2.3075	0.1659
	TRINITY_DN63126_c0_g1	-6.3800	0.0125	-1.6653	1.0000
RAN1	TRINITY_DN1990_c0_g2	1.3619	0.0227	0.9698	0.0204
MKK9	TRINITY_DN7131_c0_g1	-1.9223	0.0483	-1.2925	0.1588
EIN3	TRINITY_DN1666_c0_g1	-1.0291	0.0490	-0.6120	0.0354
ERF1	TRINITY_DN4643_c0_g1	-2.5867	0.0069	-1.4884	0.3595
	TRINITY_DN1666_c0_g1	-1.0291	0.0490	-0.6120	0.0354
CHiB	TRINITY_DN5952_c0_g1	4.2168	0.0000	3.8340	0.0000
MYC2	TRINITY_DN9581_c0_g1	2.0506	0.0102	1.5788	0.0122
PYL	TRINITY_DN3450_c1_g1	0.7346	0.7996	2.8169	0.0410
	TRINITY_DN26605_c0_g1	3.7695	1.0000	5.3066	0.0003
	TRINITY_DN4825_c0_g1	1.6540	0.3096	3.8624	0.0003
	TRINITY_DN4825_c0_g2	2.2577	1.0000	3.7482	0.0147
	TRINITY_DN4825_c1_g1	-0.1612	0.9058	1.7602	0.0474
PP2C	TRINITY_DN9486_c0_g1	1.9863	0.0010	0.4194	0.7002
MPKKK	TRINITY_DN3589_c0_g2	-2.6973	0.0020	-3.7923	1.0000
	TRINITY_DN7027_c0_g3	-2.0482	0.0263	-1.8899	0.1205
	TRINITY_DN5086_c2_g1	-2.6898	0.0067	-4.2611	1.0000
CAT1	TRINITY_DN56912_c1_g1	-8.2187	0.0001	-1.0149	1.0000
	TRINITY_DN56912_c0_g1	-2.6962	0.0336	-8.3044	0.0004
	TRINITY_DN22344_c1_g1	-6.3402	0.0406	-2.3657	1.0000
	TRINITY_DN120244_c0_g1	-5.4774	0.0248	-3.5301	1.0000
	TRINITY_DN27751_c1_g1	4.8217	0.0016	0.6103	0.7246
	TRINITY_DN69366_c0_g1	-4.9633	0.0348	0.0000	1.0000
	TRINITY_DN69366_c0_g2	4.5094	0.0478	5.8880	0.0762
	TRINITY_DN165399_c0_g1	-6.3223	0.0075	-22.0369	1.0000
	TRINITY_DN36633_c0_g1	-3.6110	0.0149	-2.0566	0.2613
	TRINITY_DN36717_c0_g1	-8.2247	0.0001	-6.7510	0.0181
PR1	TRINITY_DN6837_c1_g1	-8.4943	0.0000	-12.1077	0.0000
	TRINITY_DN20646_c0_g1	-3.0820	0.0000	-1.6175	0.2575
	TRINITY_DN6126_c0_g1	-1.8896	0.0000	-2.3846	0.1235
	TRINITY_DN6837_c0_g1	-7.2102	0.0000	-3.7717	0.0915

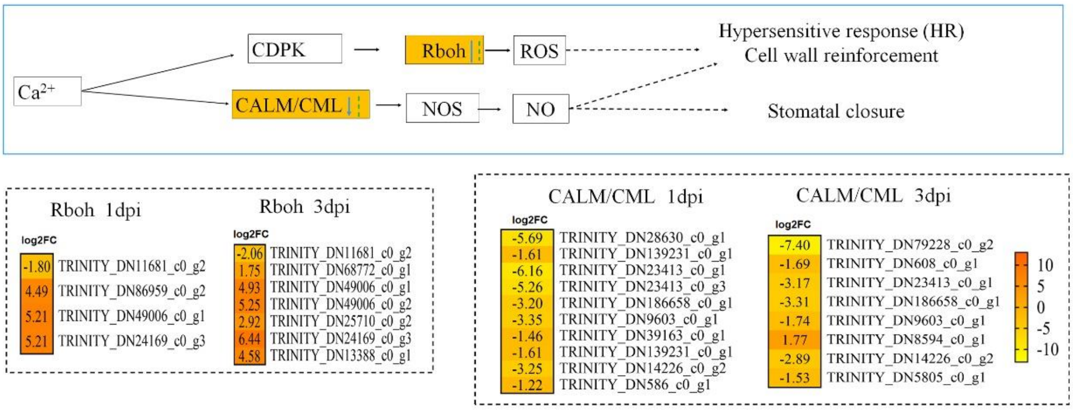

Figure S1 The difference of *calmodulin* and *RBOH* in calcium-triggered immune response.

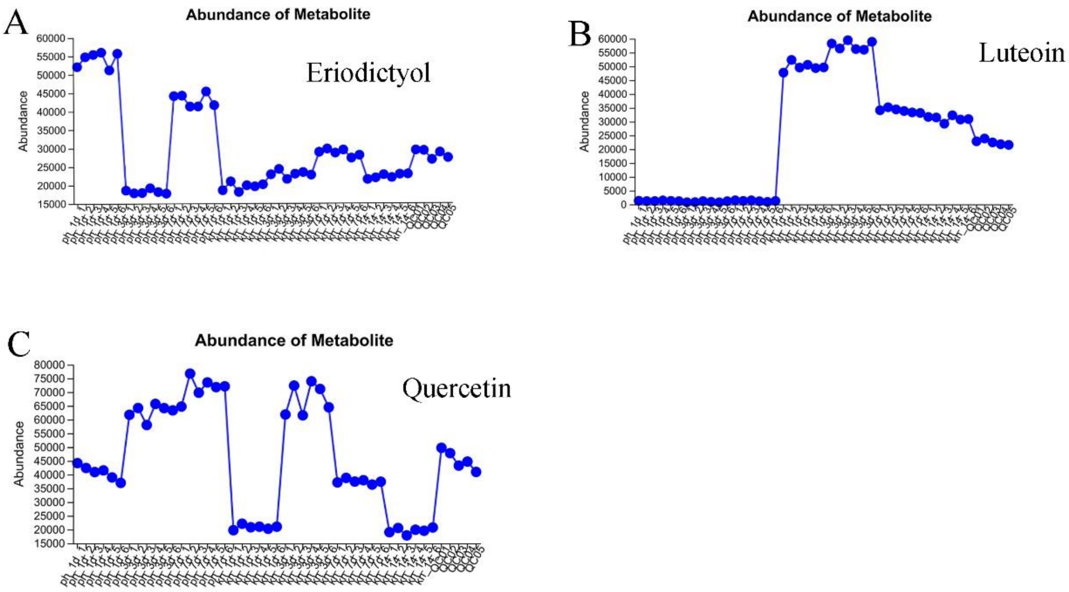

Figure S2 Abundance of metabolites in the flavonoid synthesis pathway.

ph indicates susceptible *P. thunbergii*; kh indicates resistant *P. thunbergii*. ph_1d, ph_3d and ph_7d indicate the samples (susceptible *P. thunbergii*) collected in 1, 3 and 7dpi. kh_1d, kh_3d, kh_7d and kh_14 indicate the samples (resistant *P. thunbergii*) collected in 1, 3, 7 and 14dpi. ph_1d_(1~6) and indicate biological replicates.

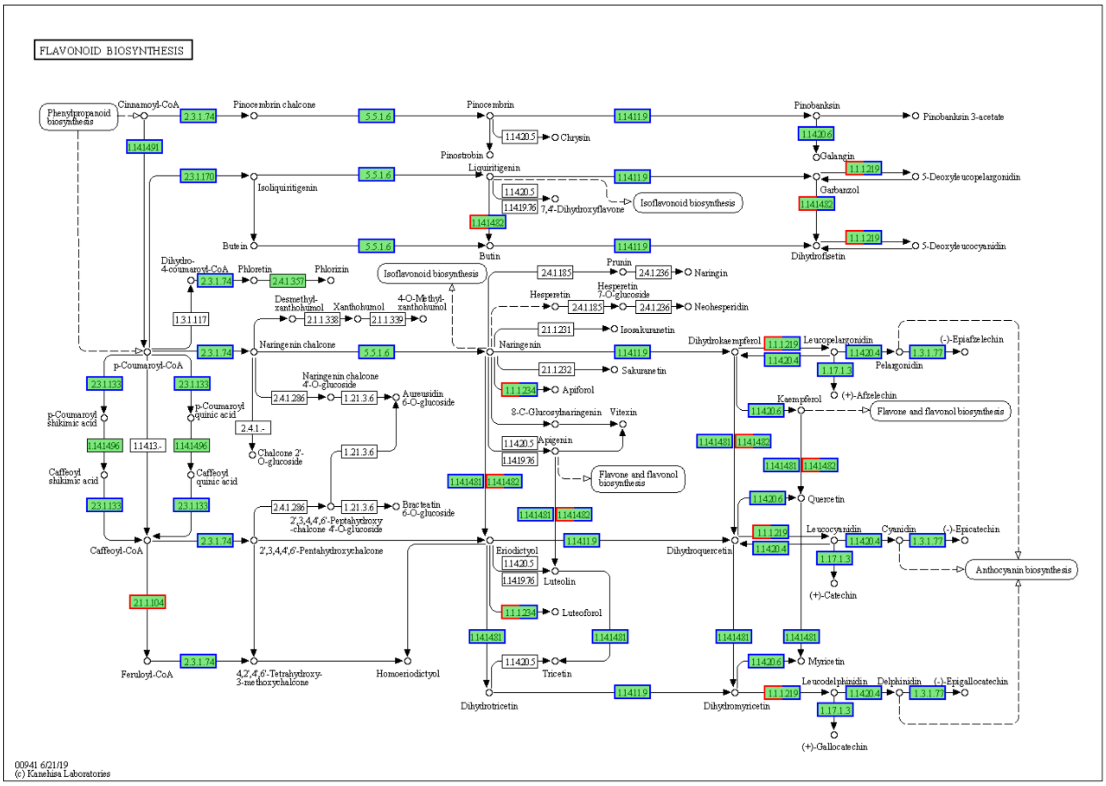

Figure S3 The difference of DEGs in flavonoid synthesis pathway at 7 dpi

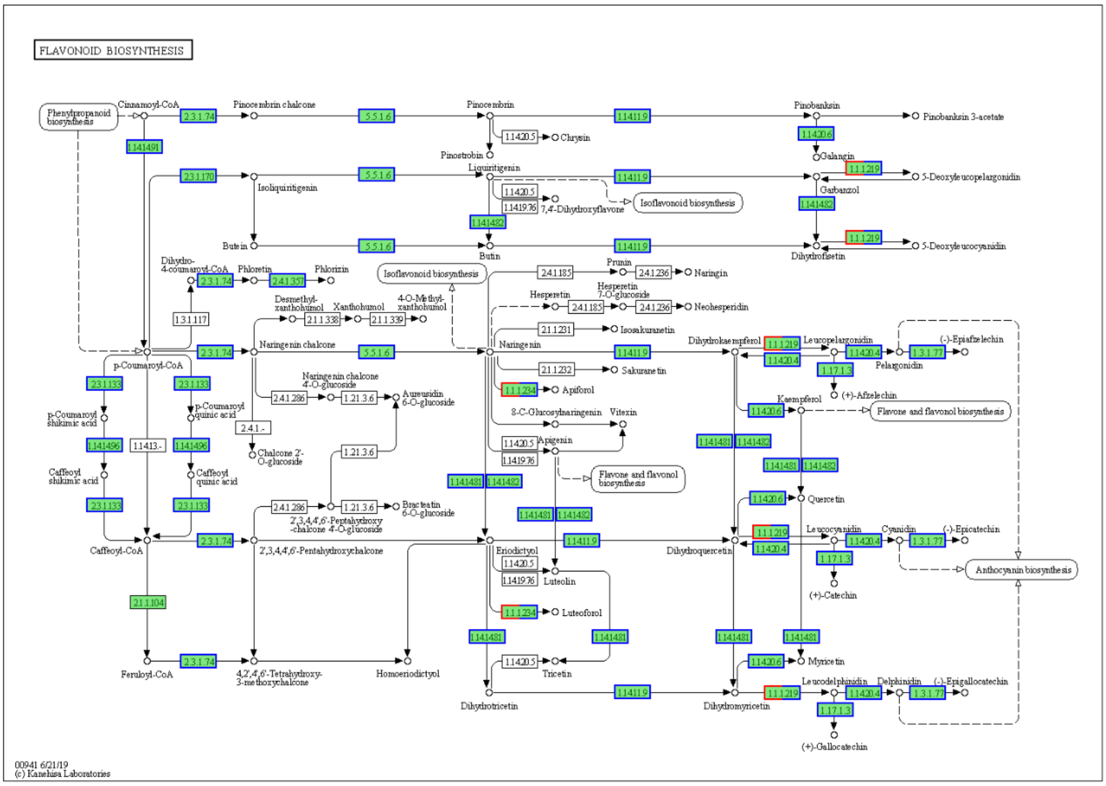

Figure S4 The difference of DEGs in flavonoid synthesis pathway at 14 dpi
